# Antiviral therapy reduces rebleeding rate in patients with hepatitis B-related cirrhosis with acute variceal bleeding after endotherapy

**DOI:** 10.1186/s12876-019-1020-2

**Published:** 2019-06-21

**Authors:** Lingling He, Xiaohui Ye, Jiali Ma, Ping Li, Yu Jiang, Julong Hu, Junru Yang, Yuling Zhou, Xiuxia Liang, Yijun Lin, Hongshan Wei

**Affiliations:** 1 Department of gastroenterology, Beijing Ditan Hospital, Capital Medical University, No.8, Jingshun East Street, Chaoyang District, Beijing, 100015 China; 2 Beijing Huaxin Hospital, the First Affiliated Hospital of Tsinghua Uinversity, Beijing, China

**Keywords:** Hepatitis virus B, Antiviral treatment, Gastroesophageal varices, Endoscopic treatment, Liver cirrhosis, Rebleeding rate

## Abstract

**Background:**

The preventive effects of antiviral therapy to reduce rebleeding rate in patients with hepatitis B-related cirrhosis undergoing endoscopic treatment have not yet been reported.

**Methods:**

In this retrospective cohort study, 1139 patients with chronic hepatitis B with first acute variceal bleeding after endoscopic therapy from September 2008 to December 2017 were included. Among them, 923 who received and 216 who did not receive antiviral therapy were followed up for rebleeding. Cumulative rebleeding rate was calculated using the Kaplan-Meier method. Univariate and multivariate logistic regression analyses were performed to estimate the effects of antiviral therapy on rebleeding risk. The propensity score matched method and inverse probability of treatment weighting analysis were used to calculate the rebleeding rate between the antiviral and non-antiviral groups.

**Results:**

The rebleeding rates were 40.5, 60.7, 72.6, and 89.2% in antiviral group at 1, 2, 3, and 5 years, respectively. The corresponding rebleeding rates in the non-antiviral group were 54.2, 72.4, 84.4, and 93.3%, respectively. The multivariate logistic regression analysis revealed that antiviral therapy was an independent protective factor associated with rebleeding.

**Conclusion:**

Antiviral treatment significantly reduced rebleeding rate in patients with HBV-related cirrhosis who received endoscopic treatment after the first variceal bleeding.

**Electronic supplementary material:**

The online version of this article (10.1186/s12876-019-1020-2) contains supplementary material, which is available to authorized users.

## Background

Chronic hepatitis B virus (HBV) infection is a critical cause of liver cirrhosis and hepatocellular carcinoma worldwide [1, 2]. As one of the regions with the highest rate of HBV infection in the world, nearly 40% of subjects with chronic HBV are living in China currently, although the vaccination strategies have decreased the number of chronic HBV patients [3]. About 350 million people worldwide suffer from chronic HBV infection, and HBV infection in China is the main cause of HCC [4, 5]. Although long-term antiviral therapy may prevent or delay the development of cirrhosis and its complications, it remains a global major public health burden, especially in China and other developing countries [6, 7]. Without antiviral therapy in patients with HBV-related cirrhosis, the 5-year cumulative incidence of cirrhosis ranges from 8 to 20%, and 5-year survival rate in those with untreated decompensated cirrhosis was low as 15%, according to the present clinical guidelines [8, 9].

Acute gastroesophageal variceal bleeding is a major complication of cirrhosis that leads to a high mortality rate (40%) and rebleeding rate (60% of survivors) [10, 11]. Therefore, prevention of bleeding in cirrhotic patients with gastroesophageal varices is one of the major therapeutic goals [12]. Most guidelines recommended that drug and endoscopic therapies should be combined for the initial treatment of acute variceal bleeding [13]. Based on a recent report, the mortality of acute esophageal variceal bleeding increases nearly up to 20% in the recent years even after using the first-line therapy, such as endoscopic varices ligation (EVL) or endoscopic varices sclerotherapy (EVS) [14]. To prevent rebleeding, several drugs were used to improve portal hypertension, in which non-selective beta-blockers (NSBBs) remained the cornerstone, albeit carvedilol seemed more effective in decreasing portal pressure [15, 16]. However, these drugs were all scrutinized in patients with severe or advanced cirrhosis [16].

Antiviral therapy was widely used to control the progression of chronic hepatitis B (CHB) during the past two decades. However, most of the current literatures focus on the immune active phases of chronic HBV infection [17, 18]; therefore, the effects of antiviral therapy on portal hypertension and gastroesophageal bleeding remain to be elucidated. In the present retrospective cohort study, we analyzed 1139 patients with CHB with acute variceal bleeding and found that antiviral therapy significantly deceased the variceal rebleeding rate, with higher survival rate.

## Methods

### Patients and design

This retrospective cohort study was conducted from 2008 to 2017 on all CHB patients with liver cirrhosis and acute variceal bleeding after an endoscopic therapy who were followed up in Capital Medical University affiliated Beijing Ditan Hospital. The exclusion criteria were as follows, i) patients coinfected with hepatitis C virus, alcoholic liver disease, and other chronic liver diseases; ii) patients with serious concurrent illness; iii) patients recurrent acute variceal bleeding or who received preventive endoscopic treatment.

These patients were chronically monoinfected with HBV who had HBV surface antigen (HBsAg)-positive for at least 6 months [19]. Liver cirrhosis was defined as the appearance of an irregular and nodular liver by two images, with impaired liver synthetic function. The normal structure of the liver lobule is severely damaged, with the evidence of the small and shrunken liver, splenomegaly and portal hypertension [20]. Acute variceal bleeding was defined as hematemesis or melena with blood pressure decreased by 20 mmHg. The study was approved by the Clinical Research Ethics Committee of Beijing Ditan Hospital.

### Clinical data collection and follow-up

Clinical data, such as age, gender, diabetes, alcohol consumption, ascites, white blood cell (WBC), hemoglobin (HGB), platelet (PLT), alanine aminotransferase (ALT), aspartate aminotransferase(AST), total bilirubin (TBIL), albumin (ALB), gamma-glutamyl transpeptidase (GGT), alkaline phosphatase (ALP), cholinesterase (CHE), creatinine (Cr), alpha fetoprotein (AFP), prothrombin time (PT), portal vein diameter, and spleen thickness, were collected at the time of acute variceal bleeding. The Child-Turcotte-Pugh score and model for end-stage liver disease (MELD) score were also recorded. The data was collected by two physicians alone, and checked by the third person.

All included patients were followed up for rebleeding and survival. The primary outcome was rebleeding rate at 1 year. Other outcomes were rebleeding rate at 2, 3, 4 and 5 years and cumulative survival rate at 1, 2, 3, 4 and 5 years.

### Antiviral and endoscopic therapy

Strategies for antiviral treatment, including administration of lamivudine, adefovir, telbivudine, entecavir, and tenofovir alone or combined, were based on the APASL guidelines and its update for the management of HBV infection [19]. Patients who received antiviral therapy before or after endoscopic treatment were all recorded.

For the endoscopic treatment of variceal bleeding, the standard EVL or EVS was performed based on the previous reports [21, 22]. Endoscopic therapies were based on Chinese guidelines: i) endoscopic variceal ligation was performed for patients with esophageal variceal bleeding; ii) cyanoacrylate was injected for the patients with gastric variceal hemorrhage; and iii) the “sandwich therapy” was used for patients with esophagealgastric variceal hemorrhage. The strategy of “sandwich therapy” was as follows: 2 ml polidocanol + 0.5 ml n-butyl-2 cyanoacrylate + 2 ml polidocanol. The injection might be repeated as necessary as previously report [23].

### Statistical analysis

The statistical analysis was performed using the SPSS version 19.0 (IBM Corp., Armonk, NY, United States) and R program (version 3.5.1, Vietna, Austria) [24]. Quantitative data were summarized with mean ± standard deviation (SD) or median with interquartile range, and their distributions in the two groups were compared using two-sample t test or Mann-Whitney U test. Qualitative data were summarized with frequency (percent) and analyzed using the chi-square test. Cumulative rebleeding and survival rates were calculated and plotted using the Kaplan-Meier method. Log-rank test was used to examine differences in rebleeding and survival rates between the antiviral and non-antiviral groups. Logistic regression analysis was performed, and odds ratio (OR) was calculated to identify variables associated with rebleeding risk. Multivariate analysis was performed with variables that showed association in the univariate analysis. Both unadjusted and adjusted ORs and 95% confidence intervals were obtained. A two-sided *P* < 0.05 was considered statistically significant.

Propensity score matching method (PSM) is associated with reduced systematic selection bias, so this method is considered as a kind of randomization for retrospective study. We used the propensity score matching method to reduce significant differences in demographics between the antiviral and non-antiviral groups. Using the multiple logistic regression analysis, a propensity score was estimated for all patients. Variables used in the model included age, sex, diabetes, alcohol consumption, ascites, WBC, PLT, HGB, ALT, AST, TBIL, GGT, ALB, Cr, PT, AFP, hepatitis B e-antigen (HBeAg), and MELD score. We performed caliper matching on the PS (nearest available matching). Pairs (antiviral and non-antiviral groups) on the PS logit were matched within a range of 0.2 SD. Rebleeding risk was calculated using the propensity score matched cohort. Inverse probability of treatment weighting (IPTW) analysis was performed to calculate rebleeding and survival rates to adjust data bias.

## Results

### Baseline characteristics

**Fig. 1:**
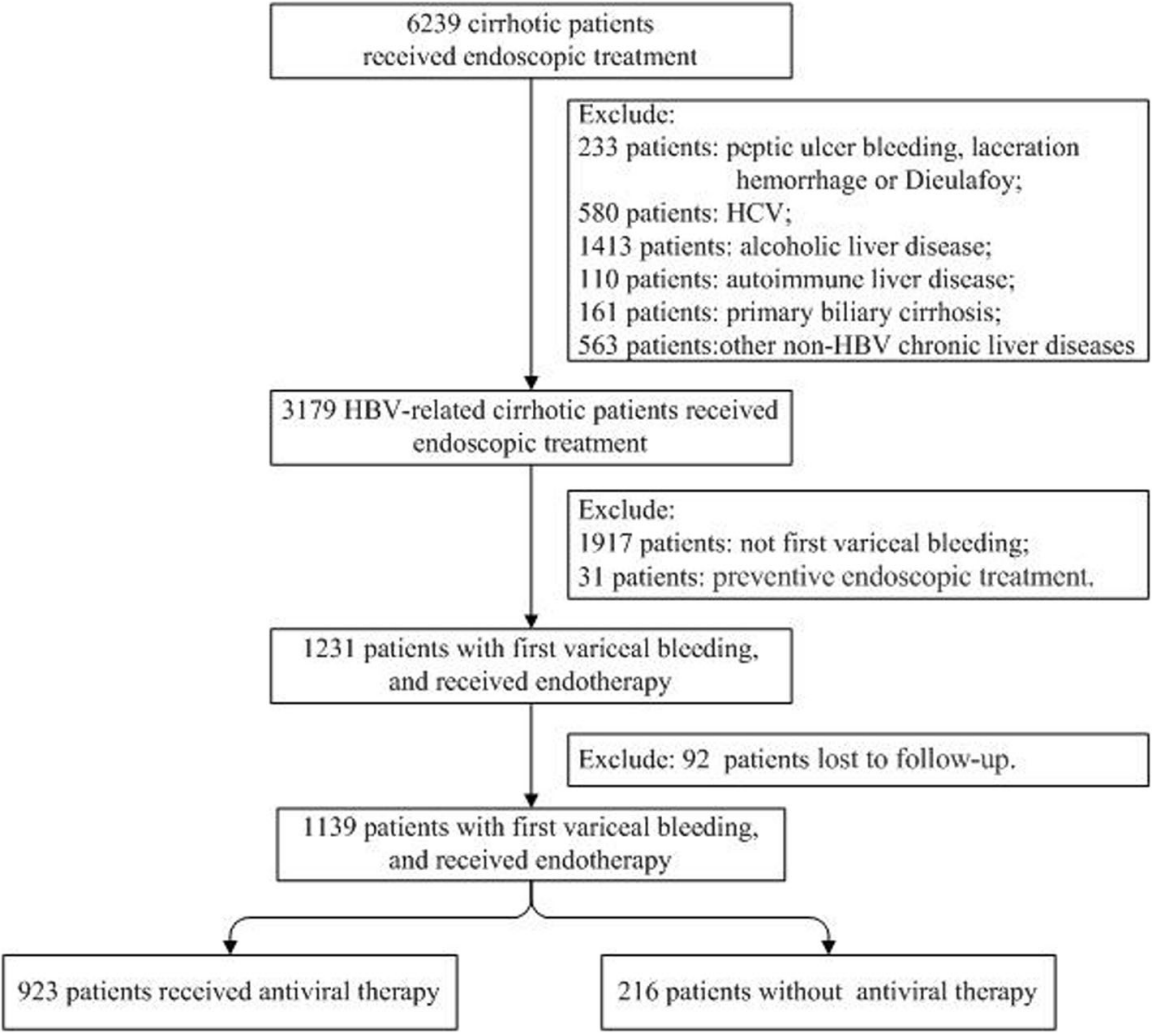
Study design

A total of 3179 patients with HBV-related cirrhosis who underwent endoscopic therapy from September 2008 to December 2017 were reviewed. After excluding patients with recurrent acute variceal bleeding (*n* = 1917), who received endoscopic treatment to prevent bleeding (*n* = 31), who were lost to follow-up (*n* = 92), the final analysis consecutively included 1139 patients with CHB with first occurrence of acute variceal bleeding and received endoscopic hemostasis. Among them, 923 patients received antiviral therapy and 216 did not (Fig. 1). The latter group consisted of patients who refused to receive antiviral medications. The patients were followed up for 1–9 years, with an average of 3.2 years.

Of the 1139 patients who were followed up over 1 year, rebleeding occurred in 491 (43.1%) after the endoscopic therapy, and 374 (40.5%) of the 923 patients who received antiviral therapy had rebleeding, whereas 117 (54.2%) of the 216 patients who did not receive antiviral therapy had rebleeding. The rebleeding rate was significantly higher in patients who did not receive antiviral therapy (*p*<0.001). The baseline characteristics are shown in Table 1. The antiviral drugs are listed in Table 2.

**Table 1:** Baseline characteristics

Characteristics	Before matching (*N* = 1139)			After matching (N = 348)		
	No rebleeding (*N* = 648)	Rebleeding (*N* = 491)	SD	No rebleeding (*N* = 192)	Rebleeding (*N* = 156)	SD
Age (yr)	50.7 ± 10.3	51.8 ± 10.4	−1.168	51.8 ± 10.6	51.8 ± 11.5	−0.019
Male sex	483 (74.5%)	364 (74.1%)	0.024	123 (64.1%)	98 (62.8%)	0.057
Antiviral therapy	549 (84.7%)	374 (76.2%)	13.291	109 (56.8%)	65 (41.7%)	7.854
Diabetes	117 (18.1%)	140 (28.5%)	17.484	19 (9.9%)	35 (22.4%)	10.324
Alcohol consumption	18 (2.8%)	16 (3.3%)	0.223	2 (1.0%)	3 (1.9%)	0.472
Ascites	479 (73.9%)	419 (85.3%)	21.824	142 (74.0%)	136 (87.2%)	9.363
WBC (× 10^9^/L)	4.0 (2.7, 6.4)	4.3 (3.0, 6.2)	−0.036	4.5 (3.0, 7.5)	4.8 (3.3, 7.0)	0.268
PLT (×10^9^/L)	62.0 (44.4, 89.0)	66.9 (46.4, 94.0)	−5.554	66.0 (44.4, 97.8)	73.5 (52.5, 111.8)	−6.968
HGB (g/l)	89.2 ± 25.2	84.1 ± 25.4	5.075	81.2 (67.4, 101.0)	77.7 (60.2, 93.2)	5.946
ALT (U/L)	23.1 (17.1, 32.8)	22.5 (17.0, 33.0)	−3.724	22.2 (17.0, 32.6)	21.7 (16.2, 33.0)	−4.208
AST (U/L)	28.6 (21.7, 39.7)	29.0 (22.4, 41.0)	−10.332	27.5 (21.6, 39.8)	28.9 (22.4, 39.5)	−13.064
TBIL (μmol/l)	17.9 (12.8, 25.3)	18.1 (11.9, 25.1)	−2.604	16.7 (11.9, 23.8)	17.1 (10.9, 24.4)	−5.402
GGT (U/L)	19.2 (12.9, 31.2)	26.4 (15.6, 45.3)	−14.324	20.1 (12.4, 33.5)	26.9 (14.1, 43.5)	−15.883
ALB (g/l)	33.1 ± 5.8	32.7 ± 5.7	0.383	31.9 ± 5.4	31.8 ± 5.7	0.163
Cr (μmol/l)	63.3 (53.7, 74.3)	63.9 (53.4, 75.8)	−0.539	58.9 (48.6, 69.4)	59.4 (48.9, 72.3)	−1.935
PT (s)	14.9 (13.9, 16.2)	15.0 (13.7, 16.4)	−0.076	15.0 (14.0, 16.5)	15.3 (14.2, 16.8)	−0.516
AFP (ng/ml)	2.8 (1.8, 5.3)	3.1 (1.7, 5.9)	−1.990	2.8 (1.8, 5.3)	3.3 (1.7, 5.9)	−0.955
HBeAg (positive)	146 (26.9%)	103 (25.6%)	0.211	21 (10.9%)	21 (13.5%)	−0.025
HBV-DNA (positive)	128 (30.3%)	86 (29.2%)	0.102	34 (26.0%)	26 (28.0%)	0.111
MELD score	6.1 ± 4.2	6.3 ± 4.9	−0.194	4.8 ± 4.8	5.7 ± 5.4	−0.881
CTP class (A/B/C)	177/359/112	81/296/114	20.548	44/113/35	20/95/41	7.386

Data are presented as mean ± standard deviation, or number (percentage). In the analysis, 946 patients detected HBeAg.718 patients detected HBV-DNA, 224 patients detected HBV-DNA after matching

*SD* standardised difference, *WBC* white blood cell, *PLT* Platelet, *HGB* hemoglobin, *ALT* alanine aminotransferase, *TBIL* total bilirubin, *GGT* gamma-glutamyl transpeptidase, *ALB* albumin, *Cr* creatinine, *PT* prothrombin time, *AFP* alpha fetoprotein, *HBeAg* hepatitis B virus e antigen, *HBV-DNA* hepatitis b virus deoxyribonucleic acid, *CTP class* Child-Turcotte-Pugh class, *MELD* model for end-stage liver disease

**Table 2:** Antiviral drugs used in patients

Drug	N(%)
Adefovir dipivoxil	188 (20.4)
Lamivudine	86 (9.3)
Entecavir	490 (53.1)
Telbivudine	9 (1.0)
Tenofovir	12 (1.3)
Adefovir + Lamivudine	77 (8.3)
Adefovir + Entecavir	43 (4.7)
Adefovir + Telbivudine	17 (1.8)
Entecavir + Tenofovir	1 (0.1)

The 1139 patients consisted of 847 men and 292 women, with the mean age of 51.2 ± 10.3 months. Patients who had rebleeding consisted of a smaller proportion from the antiviral group, had higher diabetes rate, higher ascites rate, lower HGB, higher GGT, and a large proportion of CTP class C.

In the analysis, HBV-DNA was detected in 718 patients. In patients who were HBV-DNA positive, the rebleeding rates at 1-, 3-, and 5-year follow-up were 40.2, 70.6, and 91.3%, respectively. In patients who were HBV-DNA negative, the rebleeding rates at 1-, 3-, and 5-year follow-up were 41.5, 76.3, and 92.4%, respectively. The difference in rebleeding rate between patients with positive and negative HBV-DNA was not significant. At the same time, the survival rates of patients with positive HBV-DNA at 1-, 3-, and 5-year follow-up were 94.4, 88.0, and 47.3%, respectively, whereas in those with negative HBV-DNA, they were 97.0, 87.9, and 52.3%, respectively. The difference in survival rate between patients with positive and negative HBV-DNA was also not significant.

The mean age of all patients was 51.2 ± 10.3 months. The difference in rebleeding and survival rates at 1 year based on patients’ age was not significant. However, significant differences were observed in the rebleeding and survival rates at 3 and 5 years, i.e., elderly patients were more likely to rebleed and die.

Before the propensity score matching, differences in sex, WBC, PLT, HGB, ALB, Cr, PT, AFP, HBeAg, and MELD score were significant in the entire cohort. After matching, the variables above were balanced in the propensity score matched cohort (348 patients) (Table 3).

**Table 3:** Baseline characteristics of the entire cohort and propensity score matched cohort

Characteristics	Before matching (N = 1139)			After matching (N = 348)		
	Non-antiviral group (N = 216)	Antiviral group (N = 923)	SD	Non-antiviral group (*N* = 174)	Antiviral group (N = 174)	SD
Age (yr)	51.5 ± 11.5	51.1 ± 10.1	0.336	52.1 ± 11.2	51.5 ± 10.8	0.626
Male sex	144 (66.7%)	703 (76.2%)	8.283	113 (64.9%)	108 (62.1%)	0.310
Diabetes	40 (18.5%)	217 (23.5%)	2.496	29 (16.7%)	25 (14.4%)	0.351
Alcohol consumption	6 (2.8%)	28 (3.0%)	0.040	3 (1.7%)	2 (1.1%)	0.203
Ascites	176 (81.5%)	722 (78.2%)	1.114	138 (79.3%)	140 (80.5%)	0.072
WBC (×109/L)	4.5 (3.0, 7.0)	4.1 (2.8, 6.2)	0.418	4.6 (3.1,7.2)	4.8 (3.1, 7.3)	−0.309
PLT (×109/L)	69.0 (44.4, 119.0)	63.0 (45.4, 88.4)	15.353	68.5 (44.4, 119.2)	69.0 (49.4, 96.3)	5.467
HGB (g/l)	80.0 ± 23.7	88.7 ± 25.5	−8.659	81.0 ± 23.5	82.2 ± 22.9	−1.163
ALT (U/L)	22.5 (16.8, 32.6)	22.9 (17.1, 33.0)	−3.452	22.8 (16.6, 32.9)	21.4 (16.5, 32.8)	0.464
AST (U/L)	28.4 (22.3, 41.2)	29.3 (21.9, 41.2)	−1.642	28.4 (21.8, 40.1)	28.0 (21.8, 39.4)	−0.006
TBIL (μmol/l)	18.1 (11.9, 24.9)	18.1 (12.6, 25.2)	−2.486	17.3 (11.4, 24.6)	16.1 (11.2, 23.7)	−1.240
GGT (U/L)	22.6 (12.7, 44.0)	20.7 (13.8, 35.6)	3.292	23.4 (13.1, 37.3)	21.6 (13.3, 33.5)	1.455
ALB (g/l)	31.5 ± 5.3	33.3 ± 5.8	−1.800	31.9 ± 5.2	31.9 ± 5.8	−0.039
Cr (μmol/l)	59.2 (48.5, 72.3)	64.2 (54.6, 75.4)	−2.655	58.5 (48.6, 72.1)	59.9 (48.8, 70.6)	4.031
PT (s)	15.3 (14.1, 16.7)	14.9 (13.7, 16.2)	0.332	15.2 (14.1, 16.5)	15.3 (14.0, 16.6)	−0.100
AFP (ng/ml)	2.5 (1.6, 4.6)	3.0 (1.8, 5.8)	−2.205	2.5 (1.6, 4.6)	3.3 (2.0, 5.9)	−2.293
HBeAg (positive)	18 (10.3%)	231 (30.0%)	28.471	18 (10.3%)	24 (13.8%)	0.975
HBV-DNA (positive)	28 (23.7%)	186 (31.0%)	2.492	25 (24%)	35 (29.2)	0.747
MELD score	5.6 (2.7, 8.4)	6.2 (3.9, 8.8)	−0.910	5.4 (2.6, 8.3)	5.0 (1.8, 8.2)	0.430
CTP class (A/B/C)	39/130/47	219/525/179	3.312	35/106/33	29/102/43	1.955

Data are presented as mean ± standard deviation, or number (percentage). In the entire cohort, 718 patients detected HBV-DNA, 224 patients detected HBV-DNA after matching

*SD* standardised difference, *WBC* white blood cell, *PLT* Platelet, *HGB* hemoglobin, *ALT* alanine aminotransferase, *TBIL* total bilirubin, *GGT* gamma-glutamyl transpeptidase, *ALB* albumin, *Cr* creatinine, *PT* prothrombin time, *AFP* alpha fetoprotein, *HBeAg* hepatitis B virus e antigen, *HBV-DNA* hepatitis b virus deoxyribonucleic acid, *CTP class* Child-Turcotte-Pugh class, *MELD* model for end-stage liver disease

### Factors associated with rebleeding

To estimate the effects of antiviral therapy on rebleeding risk, we performed univariate and multivariate logistic regression analyses. The univariate logistic regression analysis revealed that the antiviral therapy, diabetes, ascites, LY%, HGB, AST, GGT, CHE, AFP, and CTP class were factors associated with rebleeding. Among these factors, antiviral therapy, diabetes, ascites, HGB, and GGT were independent factors according to the multivariate logistic regression analysis (Table 4).

**Table 4:** Factors associated with rebleeding

Variable	Univariate analysis OR (95%CI)	*p*	Multivariate analysis OR (95%CI)	*p*
Age (yr)	1.011 (1.000–1.023)	0.059		
Gender	1.021 (0.781–1.336)	0.878		
Antiviral therapy	0.576 (0.428–0.777)	<0.001	0.563 (0.389–0.817)	0.002
Diabetes	1.810 (1.368–2.395)	<0.001	1.893 (1.347–2.662)	<0.001
Alcohol consumption	1.179 (0.595–2.336)	0.637		
Ascites	2.053 (1.513–2.787)	<0.001	1.820 (1.253–2.642)	0.002
WBC (×10^9^/L)	1.003 (0.969–1.039)	0.858		
LY%	0.986 (0.974–0.998)	0.026		
PLT (×10^9^/L)	1.002 (1.000–1.004)	0.122		
HGB (g/l)	0.992 (0.987–0.997)	0.001	0.994 (0.988–0.999)	0.032
ALT (U/L)	1.001 (0.999–1.004)	0.241		
AST (U/L)	1.003 (1.001–1.006)	0.012		
GGT (U/L)	1.011 (1.006–1.015)	<0.001	1.011 (1.006–1.016)	<0.001
TBIL (μmol/l)	1.004 (0.999–1.008)	0.124		
CHE (U/L)	1.000 (1.000–1.000)	0.014		
HBeAg (positive)	0.934 (0.696–1.252)	0.646		
HBV-DNA (positive)	0.948 (0.685–1.313)	0.750		
AFP (ng/ml)	1.003 (1.001–1.005)	0.007		
MELD score	1.010 (0.984–1.036)	0.474		
CTP class	1.492 (1.242–1.792)	<0.001		

*WBC* white blood cell, *LY%* percentage of lymphocytes, *PLT* Platelet, *ALT* alanine aminotransferase, *GGT* gamma-glutamyl transpeptidase, *CHE* cholinesterase, *AFP* alpha fetoprotein, *HBeAg* hepatitis B virus e antigen, *HBV-DNA* hepatitis b virus deoxyribonucleic acid, *CTP class* Child-Turcotte-Pugh class, *MELD* model for end-stage liver disease

### Benefits of antiviral treatment on rebleeding and survival

**Fig. 2:**
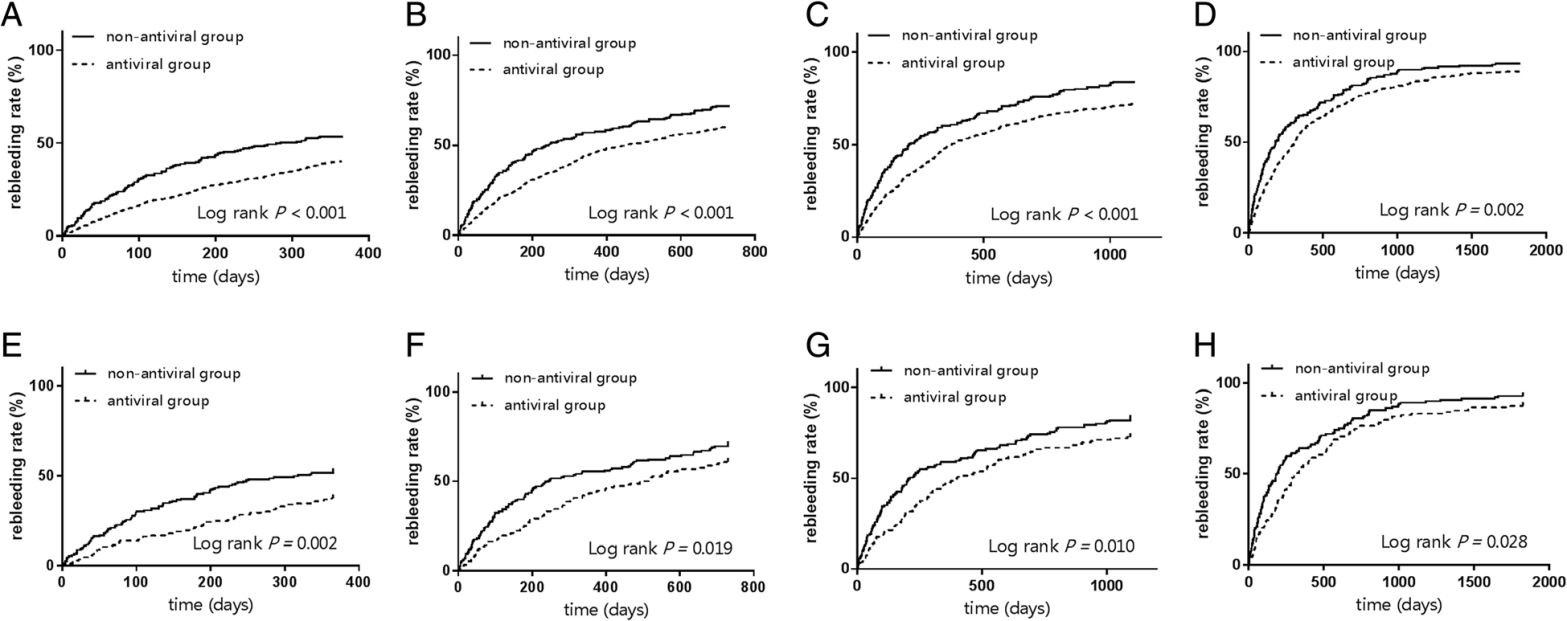
The cumulative incidence rates of rebleeding at different time points in the entire cohort (N = 1139; 216 in the non-antiviral group vs. 923 in the antiviral group) and propensity score matched cohort (N = 348; 174 in the non-antiviral group vs. 174 in the antiviral group). The entire cohort: **a**, 1 year; **b**, 2 years; **c**, 3 years; **d**, 5 years. The PS matching cohort: **e**, 1 year; **f**, 2 years; **g**, 3 years; **h**, 5 years

The cumulative incidence rates of rebleeding in different time points were analyzed. The rebleeding rate in the antiviral group was lower than that in the non-antiviral group at 1, 2, 3, 4 and 5 years (*P* <0.01) (Fig. 2a, b, c, d). The rebleeding rates were 40.5, 60.7, 72.6, 82.3 and 89.2% in the antiviral group at 1, 2, 3, 4 and 5 years, respectively. The corresponding rebleeding rates in the non-antiviral group were 54.2, 72.4, 84.4, 89.7 and 93.3%, respectively. The antiviral treatment significantly reduced the rebleeding rate.

In the propensity score matched cohort, the cumulative incidence rates of rebleeding were also lower in the antiviral group (174 patients) than that in the non-antiviral group (174 patients) at 1 (*P* = 0.002), 2 (*P* = 0.019), 3 (*P* = 0.010), and 5 years (*P* = 0.028) (Fig. 2e, f, g, h).

To further confirm the benefits of antiviral treatment, patients who received them > 1 year (*n* = 529) and < 1 year (*n* = 394) were analyzed. The cumulative incidence rate of rebleeding in patients who received antiviral treatment for more than 1 year was 37.0%, whereas it was 45.2% in patients who did not receive the antiviral treatment for 1 year (*P* = 0.023) (Additional file 1: Figure S1). Patients who received longer period of antiviral therapy had a lower incidence rate of rebleeding.

**Fig. 3:**
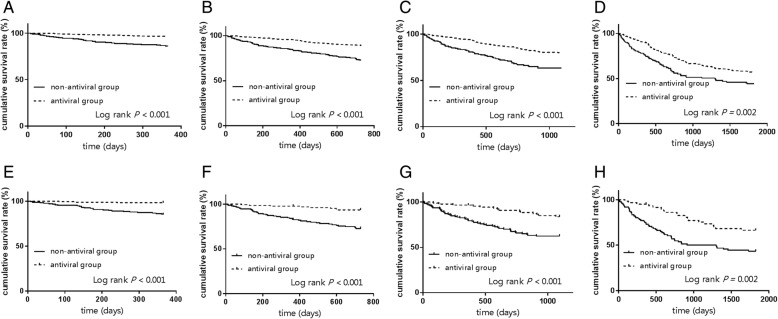
The cumulative survival rates at different time points in the entire cohort (*N* = 1139; 216 in the non-antiviral group vs. 923 in the antiviral group) and propensity score matched cohort (*N* = 348; 174 in the non-antiviral group vs. 174 in the antiviral group). The entire cohort: **a**, 1 year, **b**, 2 year, **c**, 3 year, **d**, 5 year. The PS matching cohort: **e**, 1 year, **f**, 2 year, **g**, 3 year, **h**, 5 year

We also analyzed the cumulative survival rate in different time points. The survival rate at 1, 2, 3, and 5 years in the antiviral group was higher than that in the non-antiviral group (*P* <0.01) (Fig. 3a, b, c, d). The cumulative survival rates were 96.5, 89.1, 80.6, and 59.6% in the antiviral group at 1, 2, 3, and 5 years, respectively, whereas the corresponding survival rates in the non-antiviral group were 85.6, 73.5, 64.6, and 47.2%, respectively. After matching, significant differences were still observed (*P* <0.01) (Fig. 3e, f, g, h).

**Fig. 4:**
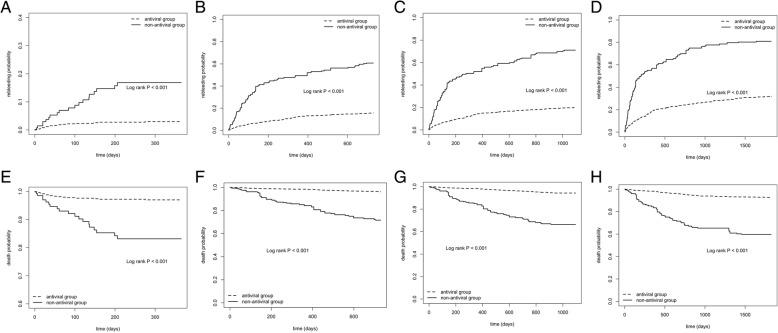
The cumulative rebleeding and survival rates at different time points by IPTW analysis (N = 1139; 216 in the non-antiviral group vs. 923 in the antiviral group). **a**, rebleeding rate at 1 year; **b**, rebleeding rate at 2 years; **c**, rebleeding rate at 3 years; **d**, rebleeding rate at 5 years; **e**, survival rate at 1 year; **f**, survival rate at 2 years; **g**, survival rate at 3 years; **h**, survival rate at 5 years

We also performed IPTW analysis to determine the benefits of antiviral treatment on rebleeding and survival rates. The result was that after the IPTW analysis, the rebleeding rate was lower and survival rate was higher in the antiviral group than those in the non-antiviral group (*P* <0.001) (Fig. 4).

## Discussion

To some extent of our knowledge, this study was the largest sample analysis to elucidate the role of antiviral therapy to prevent variceal rebleeding in patients with CHB after an endoscopic therapy.

It was widely investigated that antiviral therapy could prevent or reverse the progression of fibrosis in patients with CHB [25, 26]. Observing the 440 patients with HBV-related cirrhosis, Goyel et al. [27] found that antiviral therapy significantly improved the Child score and patient’s overall clinical course. Long-term antiviral therapy could also reduce the incidence of HCC among patients with HBV-related cirrhosis [28, 29]. A few reports focused on the effects of antiviral therapy on variceal bleeding and its outcome [30, 31]; However, no research publication elucidated the effects of antiviral therapy on patients with HBV-related cirrhosis receiving endoscopic therapy after variceal bleeding. Recently, 107 patients with HBeAg-negative compensated cirrhosis was followed up for 12 years by Lampertico and his colleagues [32]. They found that long-term antiviral therapy in HBeAg-seronegative patients with compensated cirrhosis may significantly improve preexisting esophageal varices [32]. Consistent with the above reports, our results showed that long-term antiviral therapy significantly decreased the rebleeding rate in patients with HBV-related cirrhosis after an endoscopic therapy.

The present data showed that among the factors involved in rebleeding after an endoscopic therapy, antiviral treatment was the only protective element related with decreased rebleeding rate. The results suggested that antiviral therapy should be administrated to patients with HBV-related cirrhosis with variceal bleeding, albeit receiving an endoscopic therapy. However, as a retrospective study, some natural limitations were difficult to overcome. Firstly, not all patients were periodically followed up at 3 or 6 months after the endotherapy because of economic reasons, leading to a fact that some influential factors were not analyzed in our present study. Those factors included (but not limited to) the incidence of complications and some drug-induced bias (proton-pump inhibitor, beta-receptor blocking agents, and its course of treatment). Secondly, since the varices were dynamic and might change with the endoscopic treatment (from GOV1 to GOV2), some patients might receive different endotherapy at different follow-up time, EVL, EVS, or combined therapy. Consequently, we did not analyze the relationship between the endoscopic therapies and rebleeding rate. Thirdly, as it is a retrospective study, a number of complications of HBV related cirrhosis have not been collected, such as the incidence of hepatic encephalopathy, ascites, and portal vein thrombosis. We should observe the complications of chronic HBV related cirrhosis in the prospective study in the future. Lastly, the data collected to analyze the stage of liver cirrhosis were inadequate. The liver stiffness measurement was introduced in November 2015 in our hospital, and only few patients received liver biopsy. In addition, no data were available regarding the grade of fibrosis after the therapy to analyze the study population.

Since lamivudine [33, 34], adefovir [35], entecavir [36], telbivudine [37], and tenofovir [38] all may delay or reverse liver fibrosis, we did not evaluate the differences in their efficacy. In fact, inhibiting the HBV replication is a critical factor to reverse liver fibrosis [39] and leads to decrease of the rebleeding rate in patients with HBV-related cirrhosis with endoscopic treatment.

## Conclusion

In conclusion, our present data suggested that antiviral therapy significantly reduced the rebleeding rate in patients with HBV-related cirrhosis who received endoscopic treatment after the first variceal bleeding.

## Additional file

Additional file 1: **Figure S1.** The cumulative incidence rate of rebleeding at 1 year. A, antiviral group (*N* = 923) and non-antiviral group (*N* = 216); B, antiviral treatment for > 1 year (*N* = 529) and < 1 year (*N* = 394). (TIF 126 kb)

## Data Availability

The datasets used and/or analysed during the current study are available from the corresponding author on reasonable request.

## Abbreviations

AFP: Alpha fetoprotein
ALB: Albumin
ALP: Alkaline phosphatase
ALT: Alanine transaminase
CHB: Chronic hepatitis B
CHE: Cholinesterase
Cr: Creatinine
EVL: Endoscopic varices ligation
EVS: Endoscopic varices sclerotherapy
GGT: Gamma-glutamyl transpeptidase
GOV: Gastroesophageal varices
HBV: Hepatitis B virus
HGB: Hemoglobin
IGV: Isolated gastric varices
IPTW: Inverse probability of treatment weighting
MELD: Model for end-stage liver disease
NSBB: Non-selective betablockers
PLT: Platelet
PT: Prothrombin time
TBIL: Total bilirubin
WBC: White blood cell
