# Supplementary figures and images for: Antiviral therapy reduces rebleeding rate in patients with hepatitis B-related cirrhosis with acute variceal bleeding after endotherapy
Source: BMC Gastroenterol. 2019 Jun 21;19:101. doi: 10.1186/s12876-019-1020-2 (PMC6588843; doi:10.1186/s12876-019-1020-2)

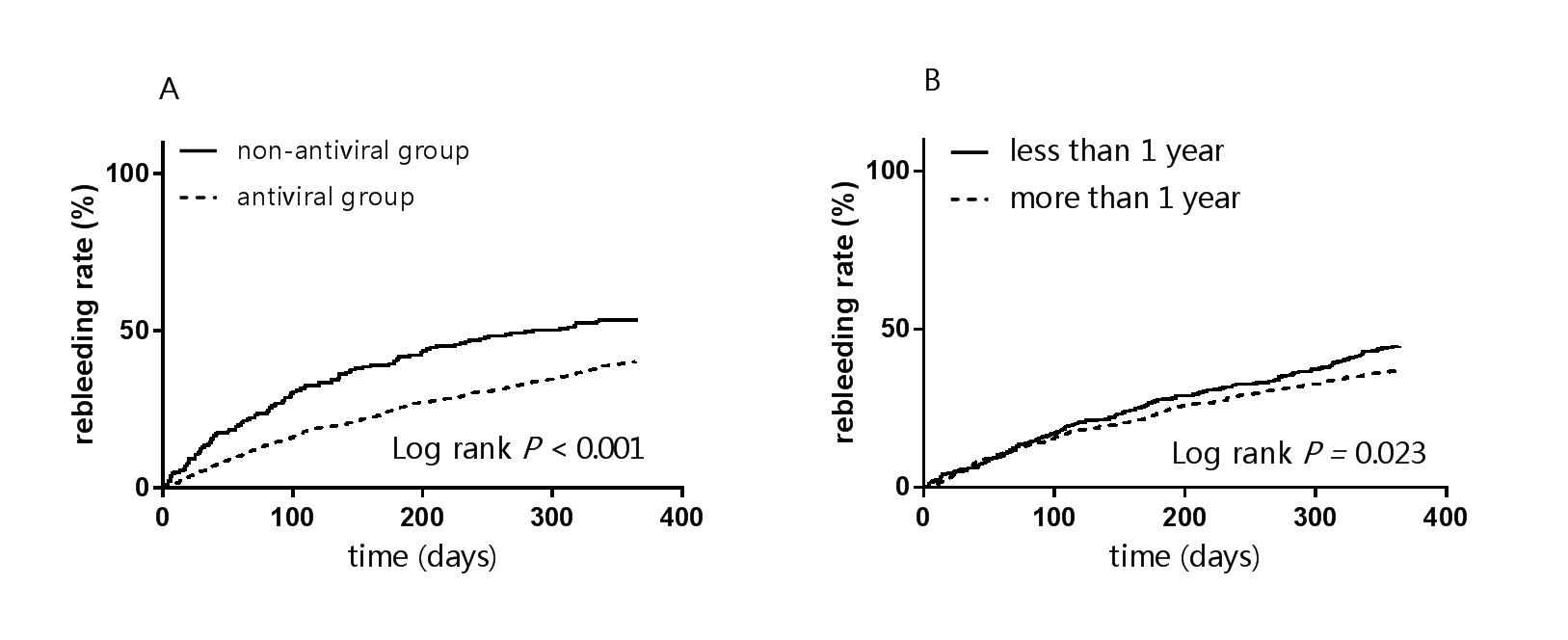

Supplement: Supplementary file 1 — Figure S1. The cumulative incidence rate of rebleeding at 1 year. A, antiviral group (N = 923) and non-antiviral group (N = 216); B, antiviral treatment for > 1 year (N = 529) and < 1 year (N = 394). (TIF 126 kb) [file 12876_2019_1020_MOESM1_ESM.tif]
